# Gender Patterns in Mobbing Victims: Differences in Negative Act Perceptions, MMPI Personality Profile, Perceived Quality of Life, and Suicide Risk

**DOI:** 10.3390/ijerph18042192

**Published:** 2021-02-23

**Authors:** Vincenzo Alfano, Tiziana Ramaci, Alfonso Landolfi, Alessandro Lo Presti, Massimiliano Barattucci

**Affiliations:** 1 Istituto Teseo, 84099 San Cipriano Picentino, Italy; studioalfanovincenzo@gmail.com; 2 Faculty of Human and Social Sciences, Kore University of Enna, 94100 Enna, Italy; 3 Department of Psychology, University of Campania “Luigi Vanvitelli”, 81100 Caserta, Italy; alfonso.landolfi@unicampania.it (A.L.); alessandro.lopresti@unicampania.it (A.L.P.); 4 Faculty of Psychology, e-Campus University, 22060 Novedrate, Italy; massimiliano.barattucci@uniecampus.it

**Keywords:** mobbing, gender, victims, workplace, suicide, quality of life

## Abstract

With the aim of investigating the impact of gender-related personality characteristics on bullying perceptions and outcomes, a correlational study was designed with 114 individuals who had used a public health service aimed at harassed workers identifying themselves as victims of mobbing in central Italy. The study was conducted using the following questionnaires: the Negative Acts Questionnaire (NAQ), a measure of workplace bullying; the Minnesota Multiphasic Personality Inventory (MMPI-2), used to provide information to measure personality dimensions for workplace screening; the World Health Organization Quality of Life (WHOQOL-BRIEF) which assesses four domains assumed to represent the quality of life construct; and the Suicidal Potential Scale (SPS) used to assess suicidal ideation. MMPI-2 profile results show a significant elevation of specific MMPI scales and gender differences. When compared to women, men who complain of being the victims of negative actions at work are more depressed, paranoid, introverted, anxious, and obsessive, and have higher anger levels and lower self-esteem. Many different MMPI-2 scales are also predictors of quality of life (QoL) perceptions and suicidal tendencies. The NAQ total score, however, predicts quality of life and suicide risk. Perceptions of negative actions have a serious effect on life outcomes. The results provide useful indications on personality profiles and gender differences, which can be understood as antecedents in the perception of negative events, and factors capable of modulating the effect of perceived bullying actions on outcomes.

## 1. Introduction

Dramatic economic and social phenomena are significantly changing working environments and related job demands, leading to parallel changes in human resource management strategies and subsequent changes in worker perceptions [1,2,3], and although there is much literature on the dysfunctional aspects of work organizations and their subsequent degenerative effects, is difficult to find a way to address clearly negative and vexatious phenomena, such as mobbing, that are instead related to personal and organizational variables [4]. Early prevalence studies suggest mobbing is a widespread and common problem in contemporary working life [1,2,3,5], and based on the epidemiological data, it can be considered one of the greatest threats to worker wellbeing [6], causing distressing consequences for both an organization and its employees [7]. 

Workplace bullying has severe negative consequences on employee health and wellbeing, organizational performance, and even social context [8,9]. Its occurrence in workplaces is high, with an estimated prevalence of 14.6% [1,2,3]. 

Even though there are overlaps and complex interrelationships among the antecedent of workplace mobbing, it is commonly stressed that this phenomenon flows from an interplay between individual and organizational predictors [10,11,12].

Different theoretical models have attempted to define the relationship between organizational characteristics, as antecedent factors (such as, for example, climate, culture, conflict management, leadership styles, etc.) and workers’ outcomes both in terms of job attitudes (commitment, satisfaction, motivation, etc.) and health (symptomatology, perceptions, quality of life, etc.) [6,13].

Mobbing is a complex phenomenon with a number of facets, which merits further academic attention [4,14] but, in any event, there are several studies that focus on the organizational perspective, with less importance placed on the possible link between harassment perceptions and a victim’s personal characteristics [15,16,17,18,19,20].

In light of this, it seems crucial to understand the various factors playing a role in the association between workplace bullying perceptions and outcomes, such as having certain personal characteristics and dispositions [21,22]. This is particularly true, given that studies on victim personality have produced conflicting evidence, with differing results found and different aspects having been assessed, [23] just as research on gender differences has often been inconsistent and unclear.

With the aim of examining the impact of gender-related personality characteristics on bullying perceptions and outcomes, a correlational study was designed with 114 individuals who had used a public health service aimed at harassed workers identifying themselves as victims of mobbing.

Building on previous studies [24] and suggestions for future research [25], the present study aimed to examine the personality profiles of mobbing victims and explore gender differences in negative action perceptions, personality profiles, and outcomes.

## 2. Mobbing and Its Effect on the Worker

Mobbing is a situation that occurs over an extended period of time, consisting of recurring negative acts that have negative effects on both victims and organizations [14,26,27]. 

Research on mobbing uses many different labels (e.g., bullying, interpersonal conflict, emotional abuse, harassment, aggression and mistreatment in the workplace, workplace victimization) [28,29,30,31], that sometimes can be used interchangeably [14].

This is an escalating situation, where the confronted individual ends up in an inferior position and becomes the target of systematically violent and deliberate acts [32,33,34], associated with negative consequences on an individual’s perceived quality of life [35]. Forms of this phenomenon may be direct, indirect, verbal, or nonverbal, and they involve “overt acts”—such as threats or actual aggression, demands for resignation, and verbal assault, or “subtle acts”—such as teasing, gossip, or banter [36]. To be considered mobbing, the situation must occur repeatedly and regularly (frequency) (e.g., weekly) and over a period of time (duration) (e.g., around six months). 

Bullying is associated with the deterioration of psychological wellbeing and increased job-related stress, which may be related with physical symptoms and psychological disorders in the victim [14,37,38] and positively predicts burnout linked to a lack of autonomy [39]. 

Different factors seem to play a buffering role in the relationship between workplace bullying and the psychological quality of life, and among these, having certain personal characteristics and personalities proved to be of significant influence [21,22,40].

## 3. Mobbing and Victim Personality Characteristics

Several studies focused on the possible relationship between personality and perceptions of negative actions at work, and on the impact on personal wellbeing and other outcomes [41,42,43,44,45,46,47], others on personality characteristics related to perceptions of being a victim or an agent of mobbing [21,41,43,44,45,48,49,50], and the level of psychological distress experienced [51]. Studies on personality differences (sense of coherence, self-efficacy, positive affectivity, negative affectivity, and self-labelling oneself as a victim) help in explaining why not all victims of bullying behaviors react to the same extent [49,52,53].

Zapf and Einarsen [54] proposed that there is no such thing as a target personality that can explain bullying in general but, in any event, personality is a key variable in determining how bullying is experienced, how it is deal with, and which personality traits seems to determine “who” in an organization is more likely to be mobbed. 

Generally, previous studies highlighted that whining, sad, rigid, prickly personalities are more frequently associated with feelings of being victimized as a result of mobbing [55,56]. Overall, available studies do not seem to clarify the role of personality differences between victims and non-victims [57]. If on one hand, some personality traits (anxiety, depression, somatization, etc.) are more frequently found in victims of mobbing [58], other studies show a rather limited role of personality characteristics in situations of conflict at work [16,59]. As far as mobbing is concerned, some studies have pointed out that mobbing can have strong negative effects on the victim’s personality and, more specifically, on their tendency to be conscientious, friendly, and open-minded [60]. Some scholars suggest that victim personality traits, therefore, could be the outcomes of negative actions perceived at work rather than the causes of the harassment suffered [61,62,63]. Although some studies have focused on the contribution and role of personality factors in relation to mobbing action effects on health consequences, very few scholars have examined the effects of mobbing on workers through personality.

The existing body of literature seems to lack empirical evidence highlighting the role of personality in predisposing a worker to be an easy target [45], and even less has focused on the analysis of the personality profile of mobbing victims through the use of Minnesota Multiphasic Personality Inventory (MMPI-2) [41,50,56,57].

Balducci et al. [59] showed that personality patterns (measured through MMPI-2) among mobbing victims present some specific features, with a tendency toward the somatization of psychological distress, as well as a notable paranoid cognition and a neurotic component. 

Another study [53] using MMPI-2 revealed that victims showed a personality profile indicating a tendency to emotional and psychological disturbance on a wide range of personality factors. However, the study showed that victims of bullying were not a homogeneous group. 

One group of victims (“seriously affected”) showed a profile indicating an extreme range of severe psychological problems and personality disturbances, although they reported a relatively low exposure to specific bullying behaviors. A second group (called the “disappointed and depressed”), showed a tendency towards becoming depressed and being suspicious of the outside world. The third group (called the “common group”), showed quite normal personalities, in spite of having experienced the highest amount of specific bullying behaviors. 

Specific vulnerability/hardiness factors may exist among some but not all victims of bullying at work. People who are already suffering from psychological problems are probably more likely to suffer long-term psychological and physical problems in the wake of bullying and serious personal conflicts. Workers with psychological problems, low self-confidence, and a high degree of anxiety in social situations may also be more likely than others to feel bullied and harassed, and they may find it more difficult to defend themselves if they are exposed to aggression by other people. 

## 4. Gender Differences in Workplace Mobbing

The experience of women in the job market has always been substantially different from that of men. As highlighted in institutional reports (Equal Opportunities Committee of the European Commission, “flex-security” in the workplace and equal opportunities), women are subjected to daily acts of persecution and violence in family and social environments, especially women who have broken the mold of traditional “working stereotypes”. There are several socio-demographic factors that seem to influence the way we perceive bullying and its effect on a person [64]. 

Results concerning gender differences have often been inconsistent and unclear. Gender-related experiences of workplace bullying may be cultural and country-specific. In some countries, no gender difference was found, whereas in a few countries, men reported being bullied more often than women at least to some extent [65,66,67,68]. Although the literature seems to lean towards men as the more aggressive gender [48,69] there are situations in which these differences dissipate, such as propensity to aggress under provocation [64]. Other studies have shown no significant relationship between aggression and gender and still others show that women are more aggressive than men [70].

Any further studies that explored gender differences in perceptions and victim reactions found that women were more likely than men to label their negative experiences as bullying [71,72,73].

More specifically, gender seems to be related to differences in both perceptions of negative actions and personality traits. In two different samples of mobbing victims, women showed a higher level of anxiety and more psychosomatic problems compared to men [40]. However, in another study [50], the results of a sample of 146 subjects showed the opposite results than those of Zapf et al. with regards to gender differences in the symptomatology measured with the MMPI-2.

In order to clarify the gender differences regarding the personality profiles of mobbing victims, the present study aims to explore the relationships between negative action perceptions, MMPI-2 personality profile, and quality of life, and to explore possible gender differences within individual clusters of workers, which is a multivariate technique that allows us to group statistical units to minimize the “internal differences” (high intra-cluster homogeneity) of each group and to maximize the external ones between the groups (high extra-cluster heterogeneity). 

The study can provide useful indications on the differing personality factors of men and women that companies should take into account for monitoring and predicting negative events and their consequences on health.

## 5. Study Aims and Hypotheses

The general purpose of this study is to examine the relationships between personality profile, negative actions at work, and perceived quality of life in employed mobbing victims, with particular reference to the influence of gender.

Specific objectives include: (a) analyzing gender differences in the general experience of bullying of workers who perceive themselves to be victims of negative actions at work; (b) exploring the association between the experience of suffering negative actions at work and an individual’s personality profile, measured through the MMPI-2; (c) analyzing the relationship between mobbing experiences and perceived quality of life; (d) analyzing the relationship between mobbing experiences and suicidal ideation.

In continuity with previous studies [59,74], a correlational study was designed for mobbing victims to explore relationships between negative actions, personality, and quality of life, and to identify any gender differences and modifications between clinical clusters. 

The relationship between mobbing and quality of life seems to be moderated by the personality traits of victims because it is believed that personality differences determine how victims react to different stress situations [60]. In that sense, it is crucial to understand the different factors that play a buffering role in the relationship between workplace bullying perceptions and life outcomes (relationship stressor–strain), such as, for example, having certain personal characteristics and personalities [21,51] that could be associated in mobbing phenomenon.

For this purpose, the research hypothesized the following: 

Hp1a—following indications in the literature, it is assumed that there are gender differences in MMPI-2 scores. The research hypothesized higher scores in both clinical and content values in men compared to women [53,59];

Hp1b—Taking into account results from previous studies, there are gender differences in the experience of being a mobbing victim. The research hypothesized higher scores related to bullying perceptions in men compared to women [24,25];

Hp2a—since different studies proved that there is a negative relationship between the perception of being victim of negative actions at work and different outcomes, it is reasonable to expect that as the total Negative Acts Questionnaire NAQ and the duration of negative actions increases, there will be a lowering of the perceived quality of life [52]; 

Hp2b—following indications in the literature, there is a positive relationship between the experience of undergoing negative actions at work and suicidal ideation. According to the hypothesis, it is reasonable to expect that with the increase in the total NAQ score and the duration of the negative actions, there will be an increase in the risk of suicide [75];

Hp3—since there is a positive relationship between the elevation of the MMPI-2 clinical scales and the deterioration of life outcomes, the study expected that with the increase of clinical scales measured with the MMPI-2 there would be a parallel lowering of the perceived quality of life [37,76,77]; 

Hp4—as there is a positive relationship between the experience of suffering negative actions at work, measured through the NAQ, and the personality profile, measured through the MMPI-2 according to the present hypothesis, it can be expected that as the total NAQ score increases along with the duration of negative actions, there will be an increase in the values of the MMPI-2 scales [53].

## 6. Materials and Methods

### 6.1. Measures 

The study was conducted using the following questionnaires:

- The Negative Acts Questionnaire (NAQ) [78,79,80]. This tool investigates the frequency of exposure to a number of mobbing behaviors and it includes 22 different types of undesirable and negative behaviors that range from indirect and subtle acts—such as gossip—to direct negative acts—such as threats or physical abuse. The NAQ’s bullying behaviors cover two categories of harassment acts: hostile acts against the person/personality of the target (e.g., spreading gossip and rumors) and hostile behaviors against the working output of the target (e.g., withholding information). In order to determine the frequency of the exposure to bullying behaviors, a 5-point Likert scale is used (1 never, 2 now and then, 3 monthly, 4 weekly, 5 daily). The respondents are prompted to state how often they have been subjected to the 22 negative acts in the questionnaire, based on their experience in their workplace, over the last six months (Cronbach’s alpha = 0.92). A further item in the NAQ explores the frequency and duration of exposure to mobbing, with the same temporal frame of reference and response categories used for the single negative behaviors described previously.
- The Minnesota Multiphasic Personality Inventory (MMPI-2) [81,82]. This test provides information to measure personality dimensions for workplace screening. There are 567 items that make up the test, with a true–false answer mode [83]. The test consists of 3 validity scales (L, F, K), plus 3 subsequent additions, 10 basic clinical scales, and 15 content scales.
  ◦ The validity scales are: the L scale (lie); the K scale (correction), to readjust by correcting the scores of the other scales; and the F scale (infrequency), to detect the presence of atypical responses. The clinical or basic scales are: Hs (hypochondria); D (depression); Hy (hysteria); Pd (psychopathic deviation); Mf (masculinity/femininity); Pa (paranoia); Pt (psychasthenia); Sc (schizophrenia); Ma (hypomania); Si (social introversion).
  ◦ The 15 content scales investigating specific clinical symptoms and problems: anxiety (ANX); fears (FRS); obsessiveness (OBS); depression (DEP); health concerns (HEA); bizarre ideation (BIZ9; anger (ANG); cynicism (CYN); antisocial behaviors (ASP); type A personality (TPA); low self-esteem (LSE); social unease (SOD); family problems (FAM); difficulty at work (WRK); difficulty of treatment (TRT).
  ◦ The following additional scales were also analyzed: the Pk scale (for post-traumatic stress), Ps scale (for symptoms related to post-traumatic stress disorder, PTSD), and the FB scale (for the elimination of uninterpretable cases).

To analyze the scores, the raw values are first converted into standard points (T points) which are compared with the standard points of a non-clinical reference standard sample. The threshold beyond which you enter the clinically relevant area of symptoms is equivalent to a score of T > 65. Scores above 60 T points indicate, in most cases, a symptomatology of moderate intensity that is not above the threshold required to be classed as clinically relevant [83].

- The World Health Organization Quality of Life (WHOQOL-BRIEF) [84] is a 24-item self-report instrument which assesses four domains assumed to represent the quality of life (QOL) construct: physical health with 7 items, psychological health with 6 items, social relationships with 3 items, and environmental health with 8 items. The WHOQOL-BRIEF questionnaire contains two items from the WHO’s overall quality of life and general health definition. WHOQOL-BRIEF provides both an overall score, relative to quality of life in general, and specific scores with respect to the four areas (Cronbach’s alpha = 0.82).
- The Suicidal Potential Scale (SPS) [85] has also been used to assess suicidal ideation. The six most direct MMPI-2 suicide-related items are items 150, 303, 506, 520, 524, and 530. These six MMPI-2 suicide-related items provided valuable information regarding suicidal ideation and behavior above and beyond that of verbal self-report. Item examples include “nobody knows, but I tried to kill myself”, “recently I thought about suicide,” ranging from “none or a little of the time” to “most or all of the time”. These items were grouped together to create a single scale, the Suicidal Potential Scale (SPS) that showed adequate internal consistency (Cronbach’s alfa = 0.71).

### 6.2. Data Analysis

A correlational research was designed with the aim of investigating the possible relation between victim characteristics, workplace harassment, and victim quality of life. 

Correlational and regression analyses were conducted, as was the analysis of variance on the MMPI-2 scales for differences in socio-demographic gender factors. The statistical software SPSS 21 was used for statistical analyses. 

Long et al. [57] and Matthiesen and Einarsen [53], reporting their work on the identification of personality profiles of mobbing victims, provided both methodological and content indications for verification of the existence of these different clusters and their interpretation. Group analysis, or cluster analysis, is a set of techniques designed to reduce the number of data, combining various data into a single group based on some “similarity” or “proximity”. In other words, an attempt is made to reduce the number of rows in the data matrix, by replacing all the rows containing the data collected in a single cluster, a data (possibly fictitious) representative of the whole cluster itself. This procedure allows “homogeneous” groups to be formed, according to a certain criterion, to which a certain number of proper characteristics to all the members of the group can be attributed (at least one characteristic must differ from group to group).

In the present study, cluster analysis was conducted through the Ward method and the measure of the Euclidean distance.

A procedure was used to eliminate cases that were among the criteria that were believed to invalidate the data relating to the MMPI-2: (a) cases with more than 29 omissions; (b) scores of >69 for the L scale; or (c) F or FB scores >99. If most of the missing values returned to the first 370 items, and the L and F scales indicated a valid protocol, the clinical and content scales were used in the statistical analyses. The K scale was used both as an indicator of validity to detect the defensive style on the test. The procedure resulted in the elimination of 10 cases that did not meet these criteria

The paper reports a study that was conducted in accordance with APA ethical standards. In line with the ethical standards of the 1964 Declaration of Helsinki, before taking part in the study, all participants were informed about the aim of the study (e.g., methods, institutional affiliations of the researcher), and were asked not to mention their name or the name of their organization anywhere in the questionnaire, to ensure privacy and anonymity; they were informed of their right to refuse to participate in the study or to withdraw consent to participate at any time during the study without reprisal; participants then confirmed that they understood the instructions well, verbally accepted the offer to participate, and began filling out the questionnaire. 

### 6.3. Sample and Procedure

Data were collected from a public health service aimed at workers in situations of employment difficulties in central Italy. The workers accessed the service between March 2016 and January 2017, asking for clinical consultations, legal advice, and psychological support. Overall, 124 workers who turned to the service participated in the survey, filling in a battery of tests preceded by a socio-demographic questionnaire. All participants filled out the entire battery of questionnaires at the end of the first appointment. The final sample considered in the statistical analysis includes 114 individuals, and it was sufficiently balanced for gender, age, marital status, and education (Table 1). 

All procedures performed in this study are in accordance with the 1964 Helsinki Declaration and its subsequent amendments or comparable ethical standards.

As for the level of education, most workers held a high school diploma (60%, *N* = 68) or a university degree (33%, *N* = 36), while a marginal share of workers had a junior high school diploma or lower (7%, *N* = 10). A large part of the sample was made up of married people (54%, *N* = 61), while around a third were unmarried (35%, *N* = 39), and 11% divorced (*N* = 14). 

Workers mainly worked in the public health sector (22%, *N* = 25) and the services and public administration sector (26%, *N* = 29). A significant share worked in the production of goods and services sector (40%, *N* = 45), while a marginal share was self-employed (12%, *N* = 15). In terms of employment contracts, most workers had an open-ended contract (80%, *N* = 91) and the share of union members was significant (20%, *N* = 23). 

## 7. Results

### 7.1. Gender Differences in Measured Variables

**Figure 1:**
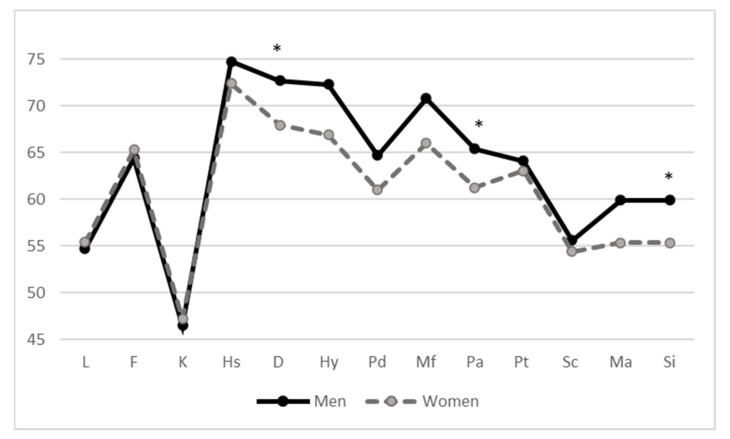
Gender differences in Minnesota Multiphasic Personality Inventory—2, MMPI-2 clinical scales (Mean T score). * significant differences of at least *p* < 0.05.

**Figure 2:**
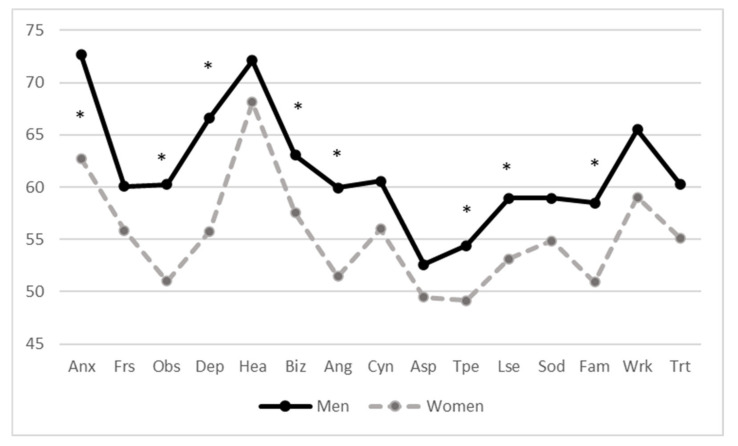
Gender differences in Minnesota Multiphasic Personality Inventory—2, MMPI-2 content scales (Mean T score). * significant differences of at least *p* < 0.05.

MMPI profiles are in line and completely superimposable on previous studies [50,53,59,80] (Table 2). In any event, univariate ANOVA highlighted significant gender differences in different MMPI-2 clinical scales (depression, paranoia, social introversion), in many content scales (anxiety, obsessiveness, depression, bizarre ideation, low self-esteem, anger, type A personality, family problems) and also in several additional scales (post-traumatic stress disorder, conjugal discomfort scale, potential drug addiction scale) (Figure 1 and Figure 2). Among the victims of negative acts in the workplace, men have, in almost all scales, average higher values than women (Table 2, Table 3 and Table 4). The Hp1a hypothesis is therefore confirmed.

No gender difference for the values of the total NAQ score, or for frequency of exposure and duration of mobbing was found (Table 5), consequently hypotheses Hp1b was not confirmed. Gender differences arose for specific dimensions of quality of life. Women have higher scores for social relation (QoL). 

### 7.2. Negative Action Effects on QoL and Suicide Risk

A regression with bootstrapping replacement was conducted to test for mediation through conditional process analysis: suicide risk was regressed using gender as a control variable, NAQ score as predictor, and QoL as a mediator [86]. Continuous variables were standardized before calculating regression models. The effect of workplace bullying was mediated by QoL, and the total explained variance was 44% (Table 6).

The Hp2a and Hp2b hypotheses are therefore confirmed. Quality of life factors, together, are also valid predictors of suicide risk.

Workers reported an average score of over three years of duration in relation to negative actions at work (average mean = 41.1 months, SD = 11.92). The duration of the negative actions expressed by the workers, however, was not significantly correlated either with the suicidal ideation or with those of the perceived quality of life. The Hp2a and Hp2b hypothesis, with regards this variable, are not confirmed.

### 7.3. Personality and Quality of Life

Different MMPI-2 scales showed a negative association with the scores of all the four dimensions of perceived quality of life: hypochondria (R^2^ = 0.07, F = 7.45, *p* < 0.01), depression (R^2^ = 0.20, F = 23.97, *p* < 0.001), conversion hysteria (R^2^ = 0.06, F = 5.81, *p* < 0.05), psychopathic deviation (R^2^ = 0.06, F = 5.93, *p* < 0.05), paranoia (R^2^ = 0.09, F = 9.61, *p* < 0.01), psychasthenia (R^2^ = 0.07, F = 6.74, *p* < 0.01), schizophrenia (R^2^ = 0.19, F = 22.92, *p* < 0.001), social introversion (R^2^ = 0.22, F = 26.73, *p* < 0.001); for content scales, anxiety (R^2^ = 0.18, F = 21.434, *p* < 0.001), frustration (R^2^ = 0.11, F = 11.88, *p* < 0.001), obsessiveness (R^2^ = 0.13, F = 14.68, *p* < 0.001), depression (R^2^ = 0.29, F = 39.33, *p* < 0.001). In any event, in women, only a few dimensions of the quality of life were predicted by some scales of the MMPI-2: psychological quality by depression (F = 6.97; β = −0.18; t = −1.09; *p* < 0.01) and psychopathic deviation (F = 5.41; β = 0.13, t = −2.33, *p* < 0.05); and environmental quality from the social introversion scale score (F = 10.53; β = 0.23, t = −3.24, *p* < 0.01). In men, on the contrary, the scores of scales of depression (R^2^ = 0.49, F = 11.58, *p* < 0.001), psychopathic deviation (R2 = 0.30, F = 5.30, *p* < 0.001), schizophrenia (R^2^ = 0.38, F = 7.56, *p* < 0.001), and social introversion (R^2^ = 0.40, F = 8.33, *p* < 0.001), were predictors of the perceived quality of life. With regards to the MMPI-2 content scales, only and exclusively in men, all dimensions of the perceived quality of life are predicted by the score of the scales of anxiety (R^2^ = 0.40, F = 8.02, *p* < 0.001), frustration (R^2^ = 0.19, F = 2.84, *p* < 0.05), and depression (R^2^ = 0.47, F = 10.94, *p* < 0.001). Many MMPI-2 clinical scales predicted suicide risk: among all, D (R^2^ = 0.55, F = 118.33, *p* < 0.001), HS (R^2^ = 0.314, F = 44.44, *p* < 0.001), HY (R^2^ = 0.23, F = 30.02, *p* < 0.001), PD (R^2^ = 0.21, F = 26.06, *p* < 0.001), and PA (R^2^ = 0.42, F = 73.20, *p* < 0.001).

### 7.4. Negative Action Perceptions and Personality

There is a direct relationship between the NAQ total score and the elevation of different MMPI-2 scales. Different MMPI-2 clinical scales are predicted by the total score of the NAQ (depression (R^2^ = 0.079, β = 0.279, t = 2.86, *p* = < 0.01), hypochondria (R^2^ = 0.04, β = 0.20, t = 2.056, *p* = < 0.05), hysteria (R^2^ = 0.05, β = 0.228, t = 2.31, *p* = < 0.05), psychopathic deviation (R^2^ = 0.10, β = 0.325, t = 3.38, *p* < 0.001), paranoia (R^2^ = 0.046, β = 0.215, t = 2.16, *p* < 0.05), and schizophrenia (R^2^ = 0.077, β = 0.277, t = 2.83, *p* < 0.01).

The comparison between men and women of the correlations between MMPI-2 scales and the NAQ total score shows substantial gender differences. For men only, numerous clinical scales (depression, hypochondria, hysteria, psychopathic deviation, paranoia, and schizophrenia) singularly correlate with the total score of the NAQ (Table 7). In relation to the MMPI content scales, for women only anger, antisocial behaviors, and workplace problems significantly correlate with the NAQ score, while in men they are the anxiety scales, health concerns, bizarre ideation, and low self-esteem (Table 8).

In the light of the gender differences highlighted by the previous analyses, the hypothesis that gender represents a mediator between perception of hostile acts at work and the personality profile and between negative actions and suicide risk was tested [24], regression analyses were computed to confirm the mediating effect of depression on the relationship between the NAQ levels and suicidal ideation. The mediation path at the Sobel test (z = 2.74, *p* = 0.006) was confirmed for the entire sample. In any event, the mediation path was not confirmed for the sample of women (z = 0.89, *p* = 0.40), but was confirmed for men (z = 3.08, *p* = 0.002).

### 7.5. Cluster Analysis

With the aim of examining the existence of different personality profiles for those who perceive themselves to be victims of bullying, a cluster analysis was carried out according to the procedure suggested by Long, Rouse, Nelsen, and Butcher [57], and starting from the database on the validity scales. Since the variables in question are measured at the level of the equivalent intervals, the cluster analysis was conducted through the Ward method and the measure of the Euclidean distance, keeping all the cases (*N* = 114).

From the inspection of the dendrogram produced by the analysis of the clusters on the 114 cases, three clusters are clearly distinguishable, corresponding, therefore, to three different personality profiles and which are made up of 33, 53, and 28 cases respectively. In Table 9 and Table 10 it is possible to observe the averages and standard deviations of the MMPI-2 validity and clinical scales in accordance with the three clusters emerging from the analysis.

In continuity with indications from the literature [53], three clusters emerged: (1) a group that does not show elevations in the validity scales (n = 33); (2) a group that, while remaining within the elevation limits, shows tendencies high on the FB scale (n = 53); and (3) a group showing a marked elevation in the scores of F and FB (n = 28).

In relation to the clinical scales, the following three clusters were generated:

(1) Cluster 1 (28% of subjects) does not show particular elevations on the validity scales and presents an elevation on the hypochondria (Hs) scale. In the study by Matthiesen and Einarsen [53] no elevation was observed in the MMPI-2 scales for the common cluster (25% of the subjects);
(2) Cluster 2 (47% of subjects) has elevations of F and FB characteristic of an appropriate expression of their symptoms, which are found in particular if F has a value between 60 and 70 T points and is greater than L and K (as in this case) [82]. This cluster shows elevations for the hypochondria (Hs), depression (D), hysteria (Hy) and paranoia (Pa) scales. The same profile emerges for the cluster of so-called “disappointed and depressed” (44% of subjects) in the study of Matthiesen and Einarsen [53];
(3) Cluster 3 (25% of subjects) presents a valid profile albeit with a marked elevation in the F and FB scales, showing a problem-oriented approach to the items of the F scale. The elevation of the FB scale could be given by a situation of severe psychopathology [83]. In this cluster there are elevations in the scales of hypochondria (Hs), depression (D), hysteria (Hy), psychopathic deviation (Pd), paranoia (Pa), psychasthenia (Pt), achizophrenia (Sc), and social introversion (Si). In the study by Matthiesen and Einarsen [53], in the cluster describes as “seriously affected” (32% of the subjects) the same elevations are highlighted, except for the scale of social introversion (Si).

For Cluster 1 there are no elevations in the content scales. The same result emerges for the analysis carried out by Matthiesen and Einarsen [53] for the “common” cluster.

For Cluster 2 (which has elevations to the limits in the norm in F and FB, and which shows elevations for the hypochondria (Hs), depression (D), hysteria (Hy), and paranoia (Pa) scales), elevations are observed in the scales of anxiety (ANX) and health concerns (HEA) In the study by Matthiesen and Einarsen [53], no elevation is observed for the content scales in the cluster called “disappointed and depressed”.

For Cluster 3 (with a definite elevation in the F and FB scales) showing elevations in the hypochondria (HS), depression (D), hysteria (Hy), psychopathic deviation (Pd), paranoia (Pa), psychasthenia (Pt), schizophrenia (Sc), and social introversion (Si) scales, there are elevations in the content scales related to anxiety (ANX), obsessiveness (OBS), depression (DEP), health concerns (HEA), ideation bizarre (BIZ), anger (ANG), and cynicism (CYN). In the study by Matthiesen and Einarsen [53] elevations are observed only in the scales of anxiety (ANX), depression (DEP), and health concerns (HEA) for the “seriously affected”.

With regards to the gender variable, it can be observed that in the first cluster there is a majority of females, while in the second and third cluster there is a prevalence of males (Table 11).

In summary, the three clusters that emerged from the Matthiesen and Einarsen study [53] and those that emerged from the present study contain results are substantially comparable, with the exception of Cluster 1, which shows a significant elevation for the hypochondria (Hs) scale, compared to the “common” group, and the elevation in the scale of social introversion (Si) found in Cluster 3, not present in the “seriously affected”.

## 8. Discussion

The research aimed to explore gender differences in personality profiles and perceptions related to negative actions at work, and their impact on perceptions of quality of life and suicide risk through a correlational study of workers who used a support service for mobbing victims.

Examining the MMPI-2 profiles of the sample of workers exposed to hostile actions, there is a substantial overlap with the profiles emerging from other research [24,59,81] showing a significant elevation of specific MMPI scales and gender differences. Compared to women, men who complain of suffering negative actions at work are more depressed, paranoid, introverted, anxious, obsessive, with greater bizarre ideation and anger levels, and with less self-esteem. Moreover, they also have higher scores on the scales of family and marital distress, and potential for drug addiction. Many different MMPI-2 scales are also predictors of quality of life perceptions and suicidal tendencies.

On the contrary, no gender differences were noted in relation to either the frequency or the duration of the mobbing actions through the NAQ. However, the NAQ total score predicted QoL and suicide risk, with perceptions of negative actions having a serious effect on life outcomes [26,38,87]

Overall, the results seem to highlight that negative actions at work (with related insecurity and perception of potential job loss) have a stronger relationship with the psychological symptoms suffered by men, who, probably because they are traditionally considered to be breadwinners, feel a greater sense of responsibility for providing for the family.

However, in reading the results, it should be noted that married workers complained of a higher degree of family problems compared to those who were unmarried, as should the fact that the male sample was made up of more married subjects than the female sample. Moreover, results should be interpreted considering that, in relation to the perception of quality of life, the men in the present sample had less satisfactory social relationships than the women. It is perhaps possible to speculate that women make more use of social support networks and relationships to address negative situations, using problem-solving skills and attempting to cope “on their own” less, which has a better impact on clinical symptoms.

The greater the perception of suffering hostile actions at work, the higher the values of different MMPI-2 scales will be. This relationship shows obvious gender differences; the relationship between the elevation of some MMPI-2 scales and the NAQ score is very evident in men, while it is much more veiled in women. Even in the content scales, men and women show different MMPI-2 scales in relation to the increase in the NAQ score, for example, in women this relates only to anger, while for men there are many scales. For example, the total NAQ score in men accounts for 28% of the variance in the psychopathic deviation and depression scale together. Overall, for many MMPI-2 scales, gender seems to act as a possible mediator/moderator between the perception of suffering hostile actions and clinical effects.

The debate remains open as to whether men who are victims of bullying have more problematic personality profiles than women, or whether the impact of negative actions is more significant on men’s psychological health.

In their study, Matthiesen and Einarsen [19] proved that negative affectivity and positive affectivity determine the variation in MMPI symptoms. This further impacts on the way people with high negative affectivity tend to view their surroundings, which is with hostility and fear. On the other hand, people with high positive affectivity see the world as a pleasurable place and these people tend to be enthusiastic and energetic.

Einarsen [88] also believes that an individual’s different personality traits can predispose them to being bullied. On the other hand, being a victim of bulling can also alter the victim’s personality in such a way that they become vulnerable to further victimization. As a result, bullying can be seen as a vicious cycle, whereby one factor leads to another and that in turn becomes a cause for further aggression.

Some findings have been noted with regard to contributing individual factors related to either the target or perpetrator, such as personality traits. Identified personality traits of targets include being relatively more introverted, anxious, conscientious, neurotic, submissive [45], less agreeable [51], and having low self-esteem [55]. These characteristics may well be linked to reportedly lower social competencies and could make targets vulnerable to bullying. Alternatively, characteristics such as conscientiousness could contribute to the behavior of targets clashing with prevailing group norms (e.g., putting in more effort or following rules more closely than the group) [42]. However, contradictory findings with regards to personality persist.

What is clear is that due to the complexity of the phenomenon, a singular portrait of a target does not exist [25,51].

## 9. Conclusions

Several papers have sought to investigate organizational and role factors that are related to adverse health outcomes and other occupational outcomes [84].

The differences in reactive symptoms to mobbing in men and women can be explained by a greater propensity in women to turn to medical and psychological support services, both in general and in relation to mobbing [10,16,84].

Based on this, it may be assumed that the higher elevations of MMPI-2 profiles in men compared to women is due to women’s ability to seek early treatment for their symptoms, while men, instead, wait until levels of psychological damage are extreme before seeking help.

One of the limitations of this study is the lack of a control group with which to make comparisons. Ideally, a control group for this type of study should be limited to subjects in the same organization or in the same office and/or sector as the bullied victim. This control group would allow us to effectively understand whether the effects identified in victims are due to an organizational approach or to other variables, and if so, of what type. So far, difficulties in recruiting a control group with these characteristics has prevented the aforementioned comparisons being made. It is, therefore, necessary to clarify that the results of this study can only be extended to similar clinical samples.

Another important limitation of the study is that the criterion variables were all self-reported data, which can evoke problems of common method variance. It would be desirable for future studies to use more objective data through real health assessments (by occupational physicians, general practitioners, etc.) and organizational measurements (e.g., absenteeism, turnover, etc.).

A further limitation to be added is that organizational variables such as leadership type, organizational culture, and how work is organized have not been taken into account. However, these variables are important in a mobbing scenario, as evidenced by previous studies [50,53,56].

The results will provide useful indications on personality profiles and gender differences which can be understood as antecedents in the perception of negative events and factors capable of modulating the effect of perceived bullying actions on outcomes. Moreover, results could provide companies with indications for the differentiated management of intervention activities in the event of perceived mobbing or stressful situations. Despite the aforementioned limitations, the results of this study are significant because they provide further confirmation of many of the theoretical models put forward in recent years by various scholars. It is research into the identification and recognition of characteristics that could help identify victims and that contributes to the knowledge necessary for developing strategies at organizational, group, and individual levels, and as such is useful for recognizing victims in the workplace and providing them with preventive help.

## Figures and Tables

**Table 1:** Sample description and gender differences.

	Gender	
	Women	Men
*N* (%)	52 (46%)	62 (54%)
Married *N* (%)	26 (51%)	37 (57%)
Degree *N* (%)	18 (36%)	18 (30%)
Mean Age (SD)	42.1 (9.78)	43.3 (8.22)

**Table 2:** Descriptive statistics of Minnesota Multiphasic Personality Inventory—2 (MMPI-2) clinical scales and gender differences.

SCALE	M (SD)	Women	Men	F
L	55.08 (9.1)	55.4 (8.2)	54.7 (9.7)	0.808
F	64.9 (7.3)	65.3 (7.2)	64.4 (7.5)	0.321
K	46.8 (5.7)	47.2 (5.8)	46.5 (5.6)	0.297
HS	73.52 (13.4)	72.44 (12.4)	74.77 (13.5)	0.776
D	70.62 (11.9)	67.97 (11.8)	72.7 (10.6)	4.383 *
Hy	69.92 (13.9)	66.94 (11.4)	72.33 (15.0)	3.81
Pd	62.91 (10.5)	61.02 (6.4)	64.72 (12.2)	3.246
Pa	53.05 (7.4)	66.02 (10.9)	70.8 (10.7)	4.756 *
Pt	68.75 (11.3)	61.28 (11.3)	65.42 (11.2)	3.325
Sc	62.4 (13.3)	63.07 (9.4)	64.12 (10.8)	0.259
Ma	55.22 (11.53)	54.4 (11.9)	55.63 (10.9)	0.288
Si	58.07 (11.6)	55.37 (11.3)	59.9 (11.2)	3.972 *

* = *p* < 0.05.

**Table 3:** Descriptive statistics of Minnesota Multiphasic Personality Inventory—2, MMPI-2 content scales and gender differences.

SCALE	M (SD)	Women	Men	F
Anx	68.75 (12.3)	62.73 (12.5)	72.77 (10.4)	15.04 ***
Frs	58.38 (11.5)	55.85 (9.6)	60.06 (12.4)	1.48
Obs	56.55 (12.6)	51.00 (12.7)	60.25 (11.2)	8.29 **
Dep	62.28 (12.8)	55.78 (10.8)	66.62 (12.3)	17.21 ***
Hea	70.53 (12.8)	68.15 (11.9)	72.12 (13.2)	1.143
Biz	60.85 (11.6)	57.55 (11.6)	63.05 (11.0)	4.416 *
Ang	56.55 (12.3)	51.48 (10.1)	59.93 (12.6)	11.52 ***
Cyn	58.74 (11.8)	55.98 (12.6)	60.58 (11.1)	1.944
Asp	51.34 (9.4)	49.45 (9.6)	52.60 (9.1)	2.050
Tpa	52.30 (9.8)	49.13 (9.3)	54.42 (9.6)	4.912 *
Lse	56.62 (10.7)	53.13 (10.3)	58.95 (10.3)	4.838 *
Sod	57.30 (11.4)	54.85 (10.1)	58.93 (12.0)	1.321
Fam	55.44 (10.7)	50.90 (9.5)	58.47 (10.4)	4.912 *
Wrk	62.91 (13.0)	59.03 (13.1)	65.50 (12.4)	2.942
Trt	58.22 (13.2)	55.13 (12.9)	60.28 (13.1)	1.402

* = *p* < 0.05; ** = *p* < 0.01; *** = *p* < 0.001.

**Table 4:** Descriptive statistics of Minnesota Multiphasic Personality Inventory—2, MMPI-2 additional scales and gender differences.

SCALE	M (SD)	Women	Men	F
Oh	50.37 (9.8)	50.14 (11.7)	50.52 (8.3)	1.884
Pk	65.90 (13.6)	59.3 (11.8)	70.3 (13.02)	15.25 ***
Mds	57.26 (11.1)	52.64 (10.3)	60.39 (10.7)	7.266 **
Aps	49.16 (10.9)	43.63 (8.5)	52.85 (10.9)	21.35 ***
Aas	53.61 (11.9)	51.95 (11.8)	54.72 (11.9)	0.724

** = *p* < 0.01; *** = *p* < 0.001.

**Table 5:** Descriptive statistics of the Negative act questionnaire (NAQ) and quality of life (QoL measures and gender differences.

	M (SD)			Difference	
	Total Sample [*N* = 114]	Women [*N* = 52]	Men [*N* = 62]	t	*p*
**NAQ total score**	64.95 (21.23)	64.17 (19.72)	65.44 (20.54)	0.081	n.s.
**Frequency of exposure to mobbing**	1.71 (0.88)	1.82 (0.76)	1.63 (0.91)	0.076	n.s.
**Duration of mobbing (in months)**	6.1 (3.7)	5.9 (3.6)	6.3 (3.9)	0.11	n.s.
**QoL total Score**	2.84 (0.78)	2.98 (0.84)	2.71 (0.73)	0.055	n.s.
**Environmental QoL**	2.89 (0.61)	3.01 (0.60)	2.82 (0.60)	0.064	n.s.
**Social relation QoL**	3.03 (0.81)	3.38 (0.73)	2.80 (0.79)	3.64	<0.001 ***
**Physical QoL**	2.5 (0.42)	2.58 (0.38)	2.48 (0.45)	0.033	n.s.
**Psychological QoL**	2.7 (0.49)	2.85 (0.46)	2.69 (0.50)	0.071	n.s.
**Suicide risk**	9.17 (4.49)	8.45 (4.61)	9.66 (4.38)	−1.32	n.s.

*** = *p* < 0.001.

**Table 6:** Regression models of suicide risk.

	Suicide Risk	
Variables	B	95% CI (LL, UL)
Gender	0.10	(−0.02, 0.24)
NAQ	0.08 **	(0.06, 0.22)
QoL	−0.21 ***	(−0.36, −0.12)
NAQ > QoL	−0.11 ***	(−0.19, −0.09)
R^2^	0.44 ***	

** *p* < 0.01. *** *p* < 0.001; LL = lower limit; UL = upper limit.

**Table 7:** Gender differences in correlations between the NAQ and MMPI-2 clinical scales.

	Hs	D	Hy	Pd	Pa	Pt	Sc	Ma	Si
NAQ women	n.s.	n.s.	n.s.	n.s.	n.s.	n.s.	n.s.	n.s.	n.s.
NAQ men	0.36 **	0.43 ***	0.37 **	0.46 ***	0.30 *	n.s.	0.39 **	n.s.	n.s.

* = *p* < 0.05; ** = *p* < 0.01; *** = *p* < 0.001.

**Table 8:** Gender differences in correlations between the NAQ and MMPI-2 content scales.

	Anx	Frs	Obs	Dep	Hea	Biz	Ang	Cyn	Asp	Tpa	Lse	Sod	Fam	Wrk	Trt
NAQ women	ns	ns	ns	ns	ns	ns	0.44 **	ns	0.39 *	ns	ns	ns	0.36 *	0.33 *	ns
NAQ men	0.30 *	ns	ns	ns	0.32 *	0.27 *	ns	ns	ns	ns	0.26 *	ns	ns	ns	ns

* = *p* < 0.05; ** = *p* < 0.01.

**Table 9:** Validity scales for the three clusters.

Cluster		L	F	K	Vrin	Trin	FB
1 *N* = 33	M SD	60.64	52.32	54.39	45.88	55.73	47.89
		8.368	7.281	9.025	7.366	3.759	4.529
2 *N* = 53	M SD	52.03	64.32	42.60	59.89	57.70	60.21
		8.628	10.283	6.625	11.862	5.523	9.106
3 *N* = 28	M SD	46.18	76.79	35.26	53.64	59.22	84.23
		6.646	12.672	3.860	6.454	8.920	12.468
Total *N* = 107	M SD	52.97	64.10	44.06	54.38	57.53	62.82
		9.655	13.532	9.857	11.180	6.256	16.283

Variable Response Inconsistency (VRIN) Scale. True Response Inconsistency (TRIN) Scale.

**Table 10:** Clinical scales for the three clusters.

Cluster		Hs	D	Hy	Pd	MF	Pa	Pt	Sc	Ma	Si
1 *N* = 33	M SD	67.53	61.81	63.05	58.83	48.40	59.33	51.93	54.83	49.90	47.87
		15.08	10.44	13.94	11.04	7.59	9.99	8.31	7.85	7.43	8.16
2 *N* = 53	M SD	72.88	71.73	71.64	64.66	50.92	69.48	63.76	63.06	53.70	59.24
		11.54	9.93	12.74	8.97	8.84	9,32	9.11	8.29	10.15	9.80
3 *N* = 28	M SD	81.19	77.55	74.12	65.26	56.63	77.74	71.78	74.93	64.11	66.70
		11.18	10.75	13.74	11.762	7.29	6.63	15.82	7.011	12.75	10.47
Total *N* = 114	M SD	73.48	70.42	69.86	63.18	51.65	68.72	62.47	63.75	55.26	57.93
		13.41	11.77	13.92	10.58	8.63	11.14	13.15	10.7	11.48	11.77

**Table 11:** Gender in different clusters.

Gender X Cluster		1 *N* = 33	2 *N* = 53	3 *N* = 28	Total *N* = 114
Men	N	9	32	21	62
	gender %	15.3	50.8	33.9	100
	cluster %	30	60	74.1	55.1
Women	N	23	21	7	52
	gender %	43.8	41.7	14.6	100
	cluster %	70	40	25.9	44.9

## Data Availability

The dataset of the study is available upon request of interested parties.
